# High-Risk Medication Use and Polypharmacy in Older Veterans Who Attempt Suicide

**DOI:** 10.1093/geroni/igaa057.2690

**Published:** 2020-12-16

**Authors:** Ruth Morin, Yixia Li, Michael Steinman, Ilse Wiechers, Amy Byers

**Affiliations:** 1 John D. Dingell VA Medical Center, Detroit, Michigan, United States; 2 Northern California Institute for Research and Education, San Francisco, California, United States; 3 University of California, San Francisco / San Francisco VA Health Care System, San Francisco, California, United States; 4 Office of Mental Health and Suicide Prevention, Department of Veterans Affairs, Menlo Park, California, United States

## Abstract

Late-life veteran suicide is a public health concern, and may overlap with recent high-risk medication use. We identified use in the 6 months prior to attempt and assessed salient risk factors. 13,872 veterans aged 50 years and older that attempted suicide were compared with demographically-matched controls utilizing VHA healthcare in a similar time period. Medications potentially related to suicide risk were included. Other variables were psychiatric and medical diagnoses, fatality of attempt and means. Compared with controls, veterans who attempted were nearly 3 times more likely to have been prescribed benzodiazepines and opioids, even when controlling for other diagnoses. Those taking 3 or more high-risk medications were between 7 and 11 times more likely to attempt than controls, with a higher risk of death particularly by drug overdose. These findings begin to uncover the complex contribution of prescription medications and polypharmacy to late-life veteran suicide, with implications for prevention. Part of a symposium sponsored by the Aging, Alcohol and Addictions Interest Group.

